# Role of non-coding RNA in exosomes for the diagnosis and treatment of osteosarcoma

**DOI:** 10.3389/fonc.2024.1469833

**Published:** 2024-10-24

**Authors:** Xin Liu, Yaling Wang, Chenwen Wang, Xinyuan Wang, Gangqiang Tang, Zhou Xiong, Wei Zhou

**Affiliations:** ^1^ Department of Orthopedics, Liyuan Hospital, Tongji Medical College, Huazhong University of Science and Technology, Wuhan, China; ^2^ Department of Nephropathy, Huanggang Hospital of Traditional Chinese Medicine, Hubei University of Traditional Chinese Medicine, Huanggang, China

**Keywords:** exosome, osteosarcoma, non-coding RNA, micro-RNA, long non-coding RNA, circular RNA

## Abstract

Osteosarcoma (OS) is a malignancy characterized by the proliferation of osteoblasts that predominantly affects pediatric and adolescent populations. At present, early detection of OS is significantly lacking, coupled with treatment challenges such as high recurrence rates, increased side effects, and the development of drug resistance. Therefore, developing new diagnostic and therapeutic modalities is clinically significant. Exosomes are naturally occurring nanoparticles found in the body that contain various materials, including DNA, RNA, and proteins. Owing to their numerous beneficial properties, including histocompatibility and *in vivo* stability, they can be useful as drug carriers. With the development of competitive endogenous non-coding RNA (ncRNA) networks, the role of ncRNA in OS cell control has been increasingly studied. This review provides a thorough summary of multiple potential biogenetic pathways of different ncRNAs in exosomes, including microRNAs, long ncRNAs, and circular RNAs. Moreover, the review highlights their effects on OS cells and their potential applications in the diagnosis, treatment, and control of OS drug resistance. The interplay between different types of ncRNAs, which collectively affect OS through the networks of competing endogenous ncRNAs, is the primary focus of this research.

## 1 Introduction

In osteosarcoma (OS), a type of osteoblastosis, tumor cells generate neoplastic osteogenesis or neoplastic osteoid matrix. The disease primarily affects children and adolescents, typically occurring proximal humerus and near the knee joint of the distal femur, proximal tibia (1–3). Currently, routine radiography and other imaging procedures, as well as the clinical manifestations of patients with frequent bone pain without induction at night, are the primary means of diagnosing OS. Additional diagnostic methods include biopsy, pathology, immunohistochemistry, and auxiliary examinations (4). Before 1970, treatment of OS primarily involved surgical resection. The current MDT strategy involves adjuvant chemoradiotherapy, surgical resection, and preoperative neoadjuvant chemotherapy using a combination of high-dose methotrexate, adriamycin, and cisplatin (5, 6). Unfortunately, these treatments have numerous disadvantages, including incomplete surgical resection, chemoradiotherapy side effects, and tumor resistance development (7).

Exosomes are extracellular vesicles with diameters ranging from 30 to 100 nm. Moreover, they are packed with various RNAs, proteins, lipids, and DNA (8). Exosome research has grown rapidly since 2012, mostly concentrating on the fundamentals of tumor-targeted therapy. With the advancement in exosome research, the non-coding RNA (ncRNA) in exosomes has attracted significant attention. This type of RNA does not translate proteins. There are numerous types of ncRNA, including miRNA, lncRNA, circRNA, tRNA, snRNA, siRNA, and piRNA (9). Exosomal ncRNAs have been explored as potential tools for the early detection and treatment of bone tumors. These ncRNAs are involved in OS cell proliferation, metastasis, apoptosis, and drug resistance (10).

However, few studies on the function of ncRNAs in exosomes in OS have discussed the interactions between different forms of ncRNAs, which collectively affect OS proliferation, metastasis, and death. This can be attributed to our inadequate knowledge of their critical role in these processes. However, they have various effects on the onset and progression of OS through synergistic or competitive antagonistic interactions. Moreover, they play a major role in determining the course of tumor treatment, including therapeutic targets and drug resistance (11). In this study, three extensively researched RNAs, circRNAs, lncRNA, and miRNAs, were chosen for evaluation. The study emphasized their roles in the diagnosis and treatment of OS, particularly their interactions that jointly affect growth, proliferation, apoptosis and drug resistance of OS cells.

## 2 ncRNA in exosomes

Exosomes are a component of intracellular vesicles, which are mainly produced through the endocytosis pathway. In the formation of multivesicular bodies (MVB), exosomes, which serve as intraluminal vesicles(ILV), are gradually formed and accumulated in endosomal sorting complexes for transport (ESCRT)-dependent pathways and ESCRT-independent pathways through multiple deslamination. Following lysosome-mediated selective transport screening and other modifications, MVB fusion with the cell membrane through exocytosis ultimately releases ILV into the body fluids (12–14). With the progress in research on exosomes, they have demonstrated significant potential in the diagnosis and treatment of OS. Exosomes have the potential to be used as OS biomarkers that could help in early and individualized tumor diagnosis. Moreover, owing to its numerous advantages, including membrane stability and biocompatibility, it can be utilized as a natural drug delivery system to pave a new path in the field of tumor-targeted therapy (15, 16).

Nucleic acids, proteins, and lipids are among the numerous components of exosomes. These vesicles are also intimately engaged in the growth, development, and spread of tumors (17). Among these are the ncRNAs that this study mentions. RNAs that aid in the expression of genes *in vivo* are known as non-coding RNAs (ncRNAs), and they can be categorized into different forms based on factors such as nucleotide length, shape, and function. These categories include miRNA, lncRNA, circRNA, tRNA, rRNA, siRNA, snRNA, piRNA, and antisense RNA (18). circRNAs, miRNAs, and lncRNAs have been thoroughly investigated. They are also closely associated with OS (19). As a result, the purpose of the three and their interactions are the main topics of this essay. First, miRNAs influence gene expression, cell division, and apoptosis by terminating translation or directly degrading the target mRNA at the microscopic level by complementary pairing with the target mRNA (20). Macro-level manifestations include the regulation of body development and growth (21), fat metabolism (22), hematopoiesis (23), and anti-inflammatory processes (24). Second, lncRNAs influence epigenetic, transcriptional translation, and post-translational modification of genes in cells, thereby affecting the cell cycle, differentiation, growth, and development of cells and the body (25, 26). Ultimately, circRNAs function as competitive endogenous RNA or miRNA sponges that regulate gene transcription and affect the cell cycle, contributing to the development and occurrence of malignancies (27) and illnesses of the cardiovascular system (28–30). More significantly, a substantial body of research demonstrates that all three of these elements are interdependent and inseparable from the body and have an impact on the OS life cycle.

## 3 Exosomal miRNAs

### 3.1 Overview of exosomal miRNAs

**Figure 1:**
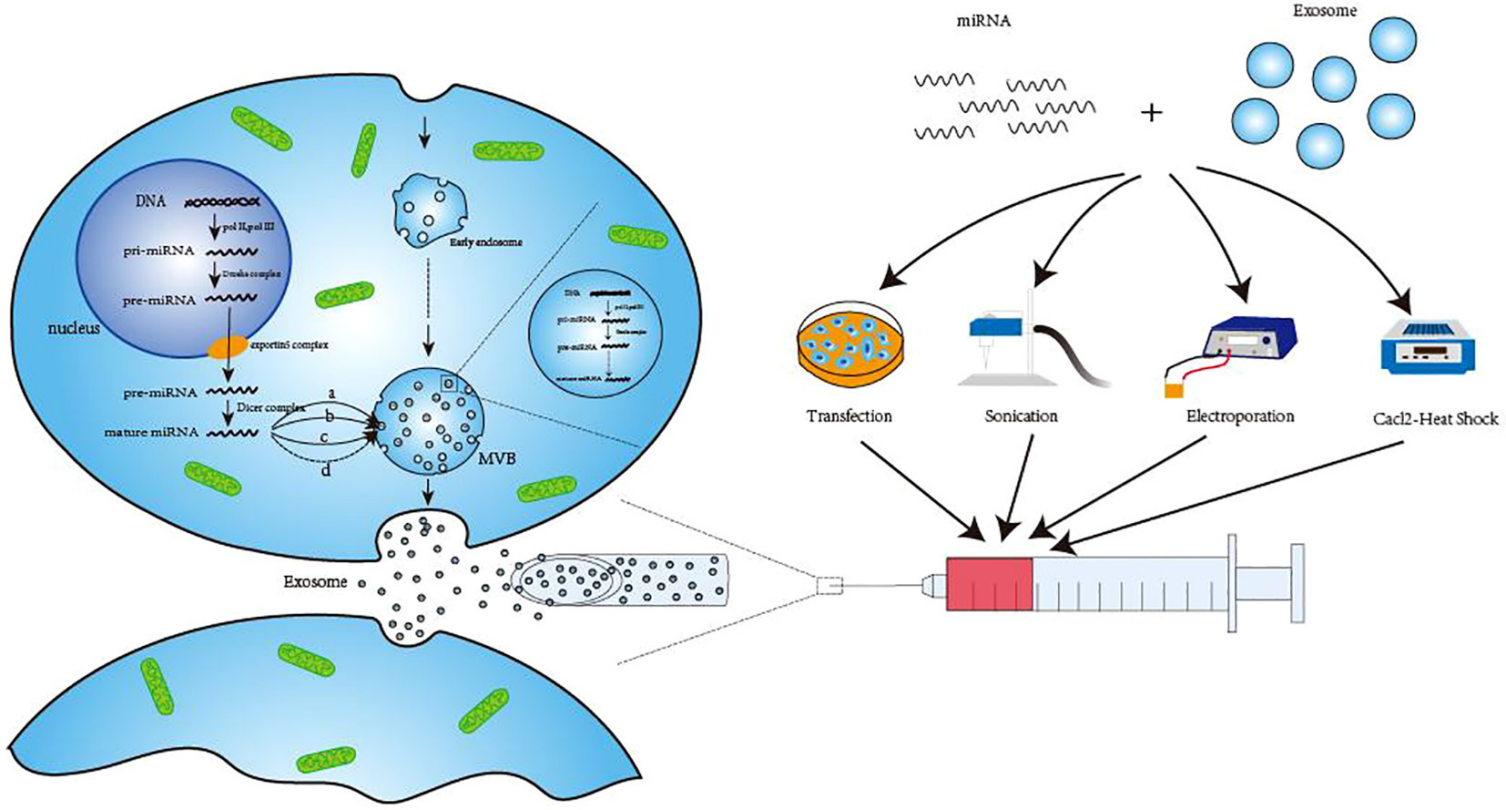
Three ways of exosomal miRNA production. The first way to generate pre-miRNA in the nucleus and then transport it to the cytoplasm to form mature miRNA after processing. Finally, hnRNP pathway **(A)**, miRNA 3’-terminal sequence-dependent pathway **(B)**, nSMase2 pathway **(C)** and possible miRNA-induced silencing complex (miRISC)/AG02 protein-related pathway **(D)** are transported to exosomes to perform corresponding functions. The second method is to use the raw material in the exosomes to directly undergo enzymatic shear processing to form mature miRNA. The third way is generated by engineering techniques, such as transfection, sonication, electroporation and Cacl-·Heat Shock. DNA, Deoxyribonucleic Acid; pri-miRNA, primary microRNA; pre-miRNA, precursor microRNA; MVB, Multivesicular body; miRNA, microRNA.

With 19–25 nucleotides, miRNAs are a type of ncRNAs that primarily assist in mRNA processing and translation *in vivo*. By destroying mRNA or regulating the initial phase of transcription, miRNAs primarily prevent their translation into proteins (31). In the natural state of the body, exosomal miRNAs are primarily formed in the following ways. Prior to maturation, the RNApol II-catalyzed translation of the miRNA-encoding gene results in pri-miRNA (19), which is further processed into pre-miRNA by the Drosha complex. Once the pre-miRNA enters the cytoplasm via the exportin5 complex (32), it gets processed by the dicer complex (33). Tang et al. (34) and Zhang et al. (35) summarized several potential sorting mechanisms in the cytoplasm that may be used to move mature miRNAs from the cytoplasm to exosomes. These include pathways that rely on particular miRNA sequences, such as the hnRNP pathways (36) and miRNA3 ‘-terminal sequence dependent pathways (37). Additionally, there are pathways that are independent of particular miRNA sequences, such as the nSMase2 pathway (38) and potential miRNA-induced silencing complex (miRISC)/AGO 2 protein-related pathways (39). When the micronucleus (MN) carrying DNA ruptures and MVB wraps the genomic DNA that is dispersed into the cytoplasm, it is transferred to exosomal vesicles during exosome formation (40, 41). This exosome-transferred DNA has the ability to encode miRNAs and other substances which could serve as targets for treatments as well as biomarkers for the identification of specific diseases (42, 43). Engineering methods such as artificial cell modification and transfection, ultrasound, electroporation, and calcium chloride heat shock (44) can also be used to artificially load miRNAs into exosomes under unnatural conditions. (**Figure 1**) Because of space constraints, these methods are not covered in detail in this study.

### 3.2 The role of exosomal miRNAs in the life cycle of OS cells

Exosomal miRNAs affect OS cells in various phases of their life cycle. According to Zhang et al., exosomal miRNAs have two primary functions. First, tumor-derived miR-21 and miR-29a can act as ligands and bind to murine TLR7. Second, they regulate intracellular target genes and bind to target cell mRNA or other receptors as ligands to facilitate intercellular communication (35). For instance, Fabbri et al. (45) discovered human TLR8 receptors, which belong to the Toll-like receptor (TLR) family, in immune cells. This in turn causes inflammation, which stimulates tumor growth and migration. As a result, miRNAs can affect OS cells at various points during their existence. First, research indicates that certain exosomal miRNAs may facilitate OS growth and multiplication. For example, Raimondi et al. (46) showed that the OS cell-derived exosomes miR148a-3p and miR21-5p could be overexpressed to significantly enhance OS activity and differentiation. Overexpression of both can stimulate the production of angiogenic factors in HUVEC, which facilitate the creation of blood vessels. Interestingly, this was not the outcome of the co-transfection. Second, exosomes containing miR-675 (47) and miR-1228 (48) can promote OS migration and invasion. Previous studies have demonstrated that a few exosomal miRNAs, including exosomes miR-208a (49), miR-221-3p (50), and miR-1307 (51), promote OS growth, metastasis, and invasion. Finally, exosomal miR-206 is a classic example of an exosomal miRNA that can cause OS cells to undergo apoptosis or suppress their ability to proliferate and spread. Zhang et al. (52) demonstrated that the targeted suppression of TRA 2B and overexpression of miR-206 can dramatically reduce the growth and metastasis of OS and induce apoptosis. The other examples of exosomal miRNAs are miR-144-3p (53), miR-15a (54), and miR-371b-5p (55).

In summary, exosomal miRNAs significantly affect the onset, growth, multiplication, metastasis, invasion, and apoptosis of OS cells. They offer a wide range of potential applications as therapeutic targets and clinical diagnostic indicators.

### 3.3 The significance of exosomal miRNAs in the diagnosis, treatment, and prognosis of osteosarcoma, as well as their role in drug resistance

Numerous studies have demonstrated a connection between aberrant miRNAs and a wide range of illnesses, including cancer, cardiovascular system disorders, and endocrine and metabolic disorders (56). *In vivo*, OS formation, proliferation, migration, and apoptosis are significantly influenced by exosomal miRNAs (57). As a result, it has a significant application value in OS diagnosis and therapy. First, Ye et al. (58) determined that patients with OS had upregulated levels of mir-195-3p, let-7i-3p, miR-92a-3p, and miR-130a-3p using high-throughput sequencing of numerous exosomal miRNAs. In particular, the expression of miR-195-3p. These exosomal miRNAs may serve as biomarkers for early diagnosis of OS. However, further investigation is required. By inhibiting BCL6 expression, exosome miR-101 produced from adipose mesenchymal stromal cells may reduce the incidence and metastasis of OS cells, as shown by Zhang et al. (59). Therefore, it is anticipated to be a useful biomarker of metastatic OS. Second, regarding therapy, McNamara et al. (60) discovered that chemotherapy medications were administered to patients with Kaposi’s sarcoma via exosomes and that exosomal miRNAs may encourage the aggregation of chemotherapy medications within the exosomes originating from tumors. This discovery raises the possibility of poisoning certain tumor cells and the surrounding tissue using chemotherapeutic medicines. In addition, the utilization of exosomes laden with chemotherapy medications might also alter tumor migration, improving the prognosis of the tumor, as demonstrated in the McNamara et al. publication. Prognosis and treatment are related, and a patient’s treatment course has a significant impact on the prognosis.

Lastly, despite minor advancements in immunotherapy and other areas, the primary treatment for OS remains the administration of methotrexate, doxorubicin, and cisplatin (MAP) as neoadjuvant chemotherapy drugs in conjunction with surgery (61, 62). As a result, tumor resistance to chemotherapeutic medications has inevitably drawn the attention of researchers and is challenging. Exosomes provide a possible solution for tumor resistance, prompting researchers to consider them because of the ncRNAs found within them. Exosomal miRNAs affect drug efflux or inactivation, metabolic regulation, tumor microenvironment, classical DNA damage repair mechanisms, and cancer cell apoptosis; therefore, they can affect tumor resistance to drugs (63, 64). Meng et al. (65), for instance, discovered that osteosarcoma cells can enhance their cisplatin resistance through autophagy and exosomal miR-331-3p secretion. Additionally, they observed that drug-resistant osteosarcoma cells can employ exosomal transmission to impart drug resistance to neighboring osteosarcoma cells.Contrary to the findings of earlier investigations on this miRNA (66), the study by Cai et al. (67) also discovered that exosome miR-143-3p was up-regulated in doxorubicin-resistant osteosarcoma cells.Yoshida et al. (68) showed that intracellular miR-25-3p increases OS medication resistance.

In conclusion, Exosomal miRNAs have a major influence on overall OS diagnosis, therapy and drug resistance, although their implementation is hampered by a lack of clarity surrounding some of the relevant mechanisms. As a result, we hope to used exosomal miRNAs as a starting point to eventually release their huge application potential.

## 4 Exosomal lncRNA

### 4.1 Overview of exosomal lncRNAs

Long non-coding RNA (LncRNAs) are RNA molecules longer than 200nt that often do not encode proteins and have conserved secondary structures (69). They share a similar biogenesis with miRNAs. First, the transcriptional splicing of genomic DNA is catalyzed by RNA polymerase II in the nucleus. Next, 5’end-capping and 3’ end-polyadenylation occur. The effectively processed lncRNA is exported to the cytoplasm by NXF1 (70, 71). The majority of lncRNAs that enter the cytoplasm are polyribosomal components, but only a small percentage are sorted into exosomes to produce exosomal lncRNAs (72). Unfortunately, the exact mechanism of this sorting process is still unknown, however, Statello et al. (73) showed that it may be connected to several RNA-binding proteins, most notably the Major Vault Protein (MVP), which is able to bind to RNA during the transfer of RNA from the cytoplasm to exosomes and from exosomes to recipient cells, thus maintaining the stability of RNA. On the other hand, exosome lncRNAs may potentially be directly transcribed by DNA in exosomes because exosomes also include DNA derived from nuclei (40),the majority of which are non-coding sections that cannot be expressed. Nevertheless, there aren’t many studies in this field currently, more research is necessary. Finally, similar to miRNAs, exosomal lncRNAs can also be produced artificially through genetic engineering methods such as transfection (74). The development of this field of study opens the door to the possibility of loading exosomal lncRNAs with medications for targeted therapy.

### 4.2 The role of exosomal lncRNA in the life cycle of OS cells

Exosomal long noncoding RNAs are present in many different cell types throughout the body, and as research progresses, more information is being revealed about their roles. It is involved in various biological processes in the human body. First, considering the inherent characteristics of lncRNAs, they can control gene expression by influencing transcriptional and post-transcriptional modification, epigenetic inheritance, and serve as a sponge for miRNAs (72, 75). Alternatively, attachment to proteins influences the functions of related proteins (76). Second, exosomal long noncoding RNAs (lncRNAs) have the ability to act as mediators in intercellular communication; Zhang et al. (77) discovered that when exosomes containing HOTAIR were added to A549 and H1299 cells, there was an increase in HOTAIR expression as well as an improvement in cell proliferation and invasion capacity. These results suggest that lung cancer exosomes mediate intercellular communication via HOTAIR. And then promote the proliferation and migration of tumor cells. Lastly, exosomal long noncoding RNA are involved in the genesis, proliferation, metastasis, and death of OS cells. Wang et al. (78), for instance, discovered that the lncRNA ELFN 1-AS1, an exosome generated from OS cells, can function as an miR-138-5p and miR-1291 sponge to cause macrophages to polarize towards the M2 type, thereby encouraging the formation of OS. Zhao et al. (79) found that by boosting the expression of the oncogenic protein ERG and preventing the ubiquitination of cancer cells, BMSC-derived exosomes loaded with lncRNA PVT1 could encourage the proliferation and migration of OS cells. By studying the miR-29/NFIA axis, Zhang et al. (80) showed that the exosomal long noncoding RNA LIR-AS1 increased the proliferation and invasion capacity of OS cells and prevented cancer cell apoptosis. Therefore, it may be a potential therapeutic target for OS. Li et al. (81) showed that exosome-related lncRNAs influence miR-153 and autophagy-related protein 5 (ATG5), which, in turn, regulate angiogenesis, migration, and autophagy in OS cells.

### 4.3 The significance of exosomal lncRNA in the diagnosis, treatment and drug resistance of OS

The primary functions of lncRNAs *in vivo* include gene expression, mRNA shearing, epigenetic regulation, cell cycle, differentiation regulation, and many other processes (82). These proteins have diverse effects on OS cell activity, proliferation, metastasis, and death. Consequently, it offers a wide range of potential applications in tumor diagnosis and treatment. Exosomal lncRNAs are mostly used as drug delivery vehicles to treat OS and as diagnostic biomarkers. For instance, Yuan et al. (83) evaluated the lncRNA DANCR content of exosomes in benign bone tumors and healthy controls and found that OS patients had considerably higher DANCR expression in exosomes, with a statistically significant difference. Consequently, in OS patients, the exosome lncRNA DANCR could be employed as a potential tumor biomarker. Huang et al. (84) created the engineered exosome cRGD-Exo-MEG 3 by transfecting lncRNA MEG3 into exosomes and altering the targeting ligand. This exosome demonstrated a noteworthy inhibitory effect on OS. This approach is expected to be useful in the clinical treatment of OS. Lastly, exosomal lncRNAs exhibit the potential for use in the treatment of tumor drug resistance. For instance, Hu et al. (85) demonstrated that exosome lncRNA ANCR expression may increase patient overall survival rates in chemotherapy-resistant OS. Furthermore, and because its blood source is readily available and non-invasive, ANCR has the potential to be a prognostic biomarker for OS patients. Additionally, Tao et al. (86) discovered that exosome lncRNA EWSAT1 might influence tumor growth by causing an increase in angiogenic factor secretion and enhancing the sensitivity of vascular endothelial cells, a process known as the “double stacking effect.”

Exosomal lncRNAs are currently under active investigation for their potential in diagnosing and treating OS, with further studies planned for the future.

## 5 Exosomal circRNA

### 5.1 Overview of circRNAs in exosomes

CircRNAs, a unique type of ncRNA with nucleotide lengths of more than 200nt *in vivo*. Its closed-ring structure allows persistent expression and resistance to degradation (87). And it is difficult to be degraded by RNA exonuclease. With advances in research, the function of exosomal circRNAs *in vivo* has drawn increasing attention. Exosomal circRNAs can be produced in three different ways, similar to the previously mentioned lncRNAs and miRNAs. First, a procedure known as “reverse splicing” occurs in the nucleus, synthesizing circRNAs. This procedure is classified into two models: lariat splicing and direct reverse splicing, depending on the sequence of circRNA cyclization and classical splicing (88). In lariat splicing, a segment comprising certain exons is spliced out first, followed by transcription of the pre-mRNA from the DNA in the nucleus. This segment then targets the 3’terminal position of the exon downstream of the disconnected mRNA and links with it. The remaining ends of the mRNA are joined end-to-end to create a cyclization, which joins the ends to produce a linear and circular double-splicing structure. The latter is achieved by coupling intermediate bases in a complementary manner to produce a Y-shaped structure. The two ends are then split, and the separated ends are linked to produce a two-part construction that is both circular and linear. RNA helicases DDX 39A and 39 B then transport the circRNA synthesized in the nucleus to the cytoplasm, and this process is dependent on the length of the RNA (27). However, the precise procedure remains unclear. Research by Li et al. (89) on the sorting of circRNAs into exosomes revealed a relationship with miR-7, suggesting that the process is mostly controlled by the miRNA content present in cells. Additionally, Dou et al. (90) discovered that this sorting process is highly intricate and may be performed by RNA-binding proteins in exosomes. However, the precise mechanism governing this process remains unknown.

Second, although exosomes are known to contain DNA and certain associated transcriptases, there is no concrete evidence that DNA transcription occurring in exosomes can directly produce circRNAs. Nonetheless, given the intracellular origin of exosomes, it is conceivable that circRNAs may be produced directly from DNA in exosomes via several intricate processes (14). Finally, exosomal circRNAs can be produced *in vitro* using artificial modification techniques such as transfection and artificial cyclization. These techniques can be used in relevant experiments and serve as prospective clinical diagnostic markers (91, 92).

### 5.2 The role of exosome circRNA in the life cycle of OS cells

Exosomal circRNAs perform various functions *in vivo*. circRNAs have the ability to control the molecular expression level of downstream target genes by acting as a miRNA sponge. For example, circRNA Rtn4-modified BMSC exosomes function as miR-146a sponges to prevent TNF-α-induced MC3T3-E1 cytotoxicity and death in mice (93).

Additionally, circRNAs can influence gene expression by regulating transcription, interacting with RNA-binding proteins, and acting as protein sponges to influence their function (94). A small fraction of circRNA is translated into proteins that control biological processes within cells (95). Furthermore, circRNAs can act as intercellular communication media by assuming the role of exosomal content in information transfer between cells. For instance, Lin et al. (96) summarized the role of exosomal circRNA in cell-to-cell communication in cancer biology. Exosomal circRNAs may play a role in the intricate intercellular communication that occurs in the tumor microenvironment between tumor, stromal, and normal cells. Exosomal circRNAs also play significant roles in OS, including drug resistance, angiogenesis, metastasis, invasion, apoptosis, and cell proliferation. It significantly affect OS cell division, migration, invasion, and apoptosis. Li et al. (97) demonstrated that circRNA circ-0000190 nanovesicles prevent cell proliferation, migration, and invasion. Additional analyses revealed that low circRNA expression is associated with tumor growth and migration. Their study showed that the expression of circ-0000190 was considerably lower in OS cell lines than in normal cells. According to Yang et al. (98), circKEAP1 can stimulate the growth and migration of tumor cells by specifically targeting exosomal miR-486-3p in OS cells, resulting in the overexpression of MARCH1. These occurrences imply that exosomal circRNAs affect the OS cell life cycle, and could serve as diagnostic markers and potential therapeutic targets for OS.

### 5.3 The role of exosomal circRNA in OS diagnosis, treatment, and drug resistance

Owing to their relatively stable ring structure and long half-life in human serum, circRNAs can serve as serum biomarkers to aid in the identification of cancers. Numerous studies have been conducted, and while its clinical applications are still in early stages, efforts to refine and promote their use are actively underway. Because exosomal circRNAs and OS cells are intimately connected, they are expected to aid in clinical diagnosis and treatment. Li et al. discovered that the content of circ-0000190 was considerably lower in the extracellular vesicles and tissues of OS patients, with the majority being encased in extracellular vesicles, as previously described. As a result, it is anticipated that this RNA will serve as a novel biomarker for OS diagnosis. Regarding therapy, exosomal circRNAs have been studied in relation to treating OS; however, owing to numerous obstacles, their useful clinical application has not yet been realized. It is conceivable that exosomal circRNAs that can impact the life cycle of OS cells will serve as therapeutic entry points in the future, and that medications targeting these characteristics may be developed for the treatment of OS. It is theoretically feasible to prevent tumor development and migration by creating tailored exosomes that block circKEAP1, a miRNA sponge known to promote the proliferation and metastasis of OS cells, as previously discussed. This would result in anti-tumor effects. Ultimately, a study by Pan et al. (99) revealed that exosomes can upregulate their receptor cells’ resistance to cisplatin by mediating exosomal circRNA circ_103801. Circ_103801 was overexpressed in cisplatin-resistant cells compared to normal MG63 cells and was abundantly present in a significant number of exosomes. This overexpression reduced the sensitivity of MG63 and U2 cells to cisplatin, increased the expression of P-glycoprotein and multidrug resistance related protein 1, and decreased apoptosis. Considering these outcomes, circ_103801 may be employed as a predictive biomarker to assess the effectiveness of chemotherapy for OS. Moreover, further studies are required to determine its usefulness in clinical settings as a target to overcome drug resistance.

## 6 Effects of miRNAs, lncRNAs, and circRNAs interactions within exosomes on OS

Since Salmena et al. (100) proposed competitive endogenous RNA (ceRNA) regulation networks, ncRNAs have received increasing attention. Gene expression and cell cycle are regulated by interactions between various types of ncRNAs. Given that exosomes originate from the cytoplasm and include a diverse array of ncRNAs, it is extremely likely that this regulatory network also exists within exosomes. Therefore, the interactions between exosomal ncRNAs, their impact on OS cell invasion and proliferation, and their use in cancer cell diagnosis and treatment are discussed in this study.

### 6.1 Interaction between miRNA and LncRNA in exosomes

As our understanding of this subject has grown, we have discovered that miRNAs and lncRNAs in exosomes are closely linked and engage in various interactions. Yin et al. (101) summarized the relationship between lncRNAs and miRNAs, and subsequent research has focused on lncRNAs acting as miRNA sponges to competitively inhibiting miRNAs (79). Various studies have discussed the effects of lncRNAs and miRNAs on OS cells and their use in diagnosis and treatment. For instance, Wang et al. (78) found that the OS cell-derived exosome lncRNA ELFN1-AS1 can upregulates CREB1 by suppressing miR-138-5p and miR-1291, promoting macrophage polarization toward M2 and ultimately enhancing OS cell biogenesis. In a different study, Zhang et al. (102) also showed that the macrophage-derived exosome lncRNA LIFR-AS1 suppresses NFIA by downregulating miR-29a, promoting cell migration and proliferation while suppressing apoptosis. According to Zhao et al. (79), exosome-coated lncRNA PVT1 suppresses miR-183-5p and raises ERG expression, thereby boosting OS cell proliferation and metastasis. According to Li et al. (81), by downregulating miR-153 and upregulating autophagy-related protein 5, the enrichment of exosome lncRNA OIP5-AS1 may block autophagy and promote the growth, migration, and angiogenesis of OS cells. As a result, it may be used as an OS therapeutic target. Guan et al. (103) discovered that cutting down lncRNA UCA1 boosted the production of miR-145, which inhibited the malignant growth of OS cells and triggering apoptosis, thereby achieving anti-tumor effects. Chang et al. (104) discovered that lncRNA linc00881, an exosome produced by OS cells, acts as an miR-29c-3p sponge, facilitating intercellular crosstalk between lung fibroblasts and OS cells, regulating associated proteases and signaling cascades, and eventually encouraging OS lung metastases (**Table 1**). These pathways merit further investigation because they may be employed as viable targets in the management of OS. A negative feedback regulatory loop exists between miRNAs and lncRNA (101). Moreover, lncRNAs control the production of miRNAs by functioning as precursors to miRNAs (105), modifying chromatin (106), and utilizing other mechanisms. This study does not delve into specifics due to constraints related to the length of the paper.

**Table 1:** Effects of the interactions between miRNAs and lncRNAs in exosomes on OS.

Exosome lncRNA	miRNA	Mechanism axis	Biological function	References
ELFN1-AS1	miR-138-5p (–)miR-1291(-)	LncRNA ELFN1-AS1/miR-138-5p、miR-1291/CREB1	Promotes the polarization of macrophages toward M2;promotes OS cells biogenesis.	(78)
LIFR-AS1	miR-29a(-)	LncRNA LIFR-AS1/miR-29a/NFIA	Promotes OS cells migration and proliferation;inhibit cell apoptosis.	(102)
PVT1	miR-183-5p(-)	LncRNA PVT1/miR-183-5p/ERG	Promotes OS cell proliferation and metastasis.	(79)
OIP5-AS1	miR-153(-)	LncRNA OIP5-AS1/miR-153/ATG5	Inhibits OS cells autophagy; promotes OS cell growth, migration, and angiogenesis.	(107)
UCA1	miR-145(-)	LncRNA UCA1/miR-145/Wnt/β-catenin pathway	Promotes the malignant growth of OS cells;inhibits OS cells apoptosis.	(103)
Linc 00881	miR-29c-3p(-)	LncRNA Linc 00881/miR-29c-3p/MMP2/NF-κB pathway	Promotes OS lung metastases.	(104)
XIST	miR-21-5p(-) miR-375-3p(-)	LncRNA XIST/miR-21-5p/PDCD4; LncRNA XIST/miR-375-3p/AKT/mTOR signaling pathway	Inhibits OS cell proliferation and mobility;Promotes cell growth and autophagy; inhibits cell apoptosis.	(108) (109)
Linc 00852	miR-7-5P(-)	LncRNA Linc 00852/ miR-7-5p/AXL	Promotes tumor growth and metastasis.	(110)
CASC15	miR-338-3p(-)	LncRNA CASC15/miR-3383p/RAB14	Promotes OS cell growth and metastasis.	(111)

### 6.2 Interaction between miRNA and circRNA in exosomes

The discovery revealing that circRNAs function as miRNA sponges with competitive inhibitory properties (112) has sparked a rapid increase in circRNA research. The main linkage between miRNAs and circRNAs involves circRNAs acting as miRNA sponges to inhibit miRNA activity and regulate the expression of downstream target genes. This mechanism can be utilized in diverse ways for tumor diagnosis and treatment, indirectly influencing osteosarcoma proliferation, metastasis, and apoptosis. Li et al. (113) discovered that through complimentary binding with miR-29c-3p, the circRNA hsa_circ_0001564 may suppress OS cell proliferation, end the cell cycle, and cause cell death. In the circ_0009910/miR-449 a/IL 6R axis, Deng et al. (114) demonstrated that circ_0009910 upregulates the mRNA expression of the IL-6R gene by suppressing miR-449. This, in turn, alters the OS cell cycle, thereby encouraging the proliferation of cancer cells and preventing their apoptosis. Therefore, circ_0009910 may be a viable target for practical assistance in the diagnosis and treatment of OS. According to Jin et al. (115), circ-0016347 functions as an miR-214 sponge to control the expression of caspase-1, thereby enhancing OS cell invasion and proliferation. Yang et al. (98) demonstrated that circKEAP1 functions as an exosomal miR-486-3p sponge and indirectly upregulates miR-214 expression. This promotes the growth of OS cells; thus, upregulating the expression of miR-486-3p or downregulating the expression of circKEAP1 can prevent the growth of OS and stop its cell cycle (**Table 2**). However, additional attention is required because what is known in this field is insufficient, and there are currently few relevant clinical application transformations.

**Table 2:** Effects of interactions between miRNAs and circRNAs on OS.

Exosome circRNA	MiRNA	Mechanism axis	Biological function	References
hsa_circ_0001564	miR-29c-3p(-)	circRNA hsa_circ_0001564/miR-29c-3p	Promotes OS cell proliferation; inhibits cell apoptosis.	(113)
circ_0009910	miR-449 a(-)	circRNA circ_0009910/miR-449 a/IL 6R	Promotes OS cell proliferation and inhibits cell apoptosis.	(114)
circ-0016347	miR-214(-)	circRNA circ-0016347/miR-214/caspase-1	Promotes OS cell invasion and proliferation	(115)
circKEAP1	miR-486-3p(-)	miR-486-3p/circKEAP1/MARCH1	Promotes OS cell growth and progression	(98)

### 6.3 Interaction between LncRNA and circRNA in exosomes

As previously mentioned, circRNAs are a unique class of lncRNAs that function as miRNA sponges and interact with miRNAs to regulate metabolism and gene expression. Through RNA–RNA interactions, lncRNAs and circRNAs collectively establish a competitive endogenous RNA network with miRNAs and mRNAs to control apoptosis, cell metabolism, and body growth and development (116). Research on the regulatory pathways between circRNAs and lncRNAs in the OS cell cycle is limited, and the underlying mechanisms are still unknown. But we can hypothesize that, in ways similar to that of miRNAs, lncRNAs can also impact their function *in vivo* by functioning as circRNAs sponges and so influencing the OSc cells’ life cycle. Conversely, circRNAs has the same effect on lncRNAs as described above.

### 6.4 lncRNA/circRNA-miRNA-mRNA axis in exosomes

As previously discussed, research in this field has steadily grown since the introduction of competitive endogenous RNA networks. The two primary axes are circRNA-miRNA-mRNA and lncRNA-miRNA-mRNA. Through various mechanisms, they affect treatment resistance, tumor growth, metastasis, the tumor microenvironment, and cell cycle events such as OS cell proliferation and apoptosis. Wang et al. (117) demonstrated that the Notch signaling pathway, PI3K/AKT pathway, JNK and Wnt pathways, SNHG20/miR-139/RUNX2 axis, and other signaling pathways are various signaling pathways through which the lncRNA-miRNA-mRNA axis can influence the growth and metastasis of OS. For instance, by blocking miR-195-5p, lncRNA SNHG12 enhances Notch2 expression and promotes the development and spread of OS (118). As an miR-33a-5p sponge, lncRNA DANCR promotes the AKT signaling pathway, upregulates the expression of AXL, and accelerates the development of OS (119). The miR-39/RUNX2 axis can be targeted by the downregulation of lncRNA SNHG20, which can also trigger the mitochondrial apoptosis pathway, limit OS development, and cause OS apoptosis (120).

**Figure 2:**
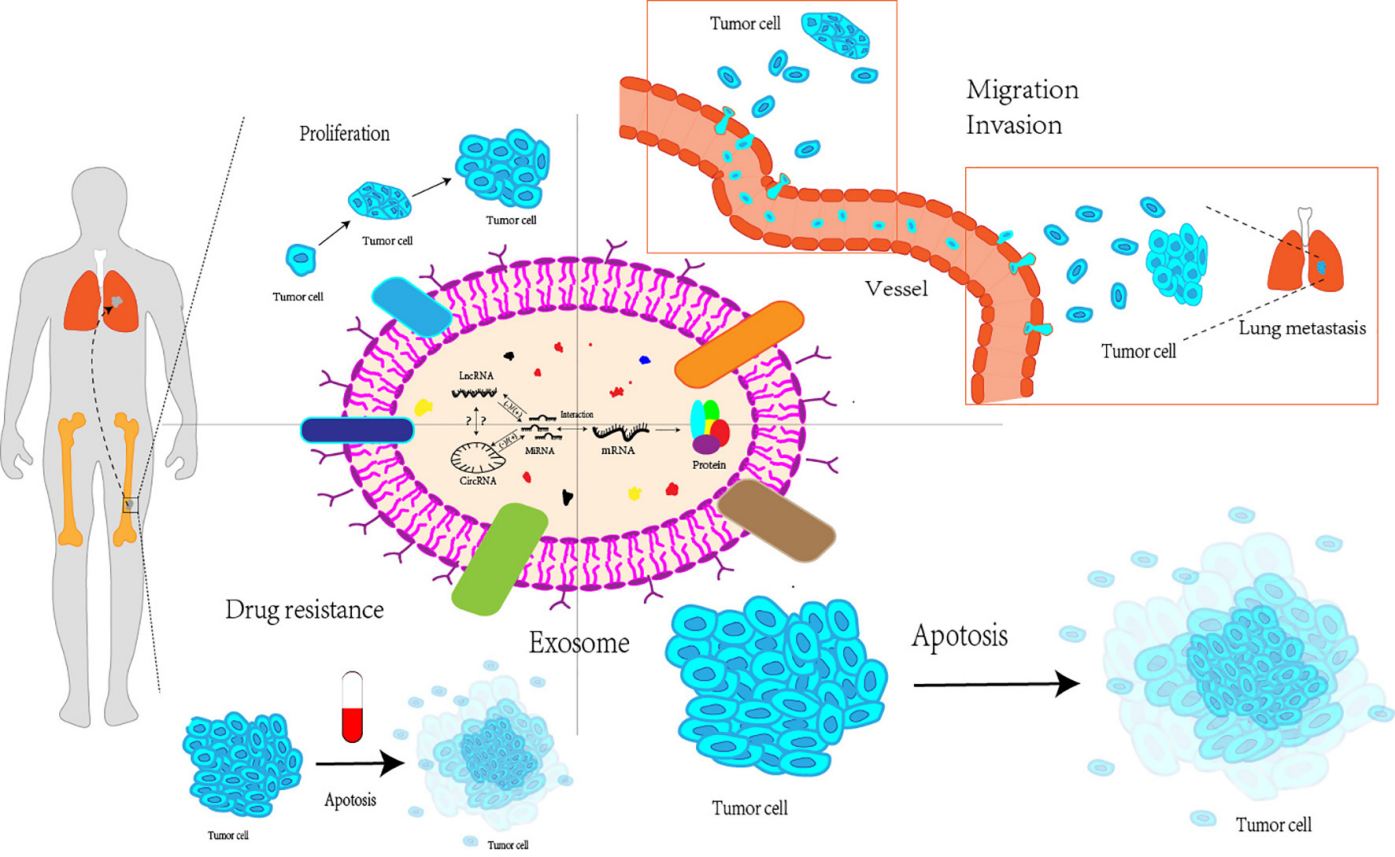
Exosomal miRNAs, lncRNAs and circRNAs interact with each other to affect proliferation, migration and invasion, apoptosis and drug resistance of osteosarcoma cells. Exosomal miRNAs, lncRNAs, and circRNAs can interact with each to affect various processes of the life cycle of osteosarcoma cells, including their proliferation stage, invasion and metastasis to distant regions, apoptosis, as well as the resistance of osteosarcoma cells to multiple drugs LncRNA, Long non-coding RNA; CircRNA, Circular RNA; MiRNA, MicroRNA; mRNA, messenger RNA.

Additionally, the circRNA-miRNA-mRNA axis affects the growth, metastasis, and treatment resistance of OS cells. Liu et al. (121) discovered that by influencing the three axes of miRNA-TRIM21, MAP3K5, and PRKX, the downregulation of circ_0001060 may ultimately decrease the proliferation and metastasis of OS cells. In their research, Qin et al. (122) concluded that competitive endogenous RNA pathways can impact tumor resistance to chemotherapeutic treatments such as doxorubicin and cisplatin through various signaling pathways, including the Wnt/β-catenin pathway and the miRNA-RASSF6 axis. In summary, competitive endogenous RNA networks have a significant impact on OS, and further research is essential to explore their potential clinical applications (**Figure 2**).

## 7 Interactions of other ncRNAs and their significance in the diagnosis and treatment of OS

ncRNAs encompass not only the three RNA types mentioned above but also siRNAs, piRNAs, snoRNA, tRNA, and long mRNA-like ncRNAs. These RNAs can also influence other RNAs, such as miRNAs and lncRNAs, to change the course of the OS cell life cycle. The characteristics and applications of siRNAs, piRNAs, and snoRNAs are briefly described in the following section.

First, cells create a class of double-stranded RNA called small interfering RNA (siRNAs), which are 21–25nt in length. Dicer nuclease cuts RNA and depends on the Argonaute family of proteins to accomplish its function (123). It controls the post-transcriptional degradation of mRNA generated by target genes. Zhao et al. (124) synthesized biomimetic nanoparticles by co-polymerizing survivin siRNA and doxorubicin hydrochloride into tumor cells. They observed that these nanoparticles had good tumor-targeting properties, were safe *in vivo*, and possibly have antitumor therapeutic effects *in vivo*. After producing chemically altered siRad 18 loaded with engineered RGD-exosomes (RGD-EXOs), Du et al. (125) discovered that lowering the expression of Rad 18 significantly boosted the susceptibility of OS cells to adriamycin. By altering the host liver, Yu et al. (126) were able to transfer small cell vesicles containing VEGFR2 siRNA to the lung. By silencing the VEGFR2 gene, siRNA decreased OS cell lung metastasis. Moreover, siRNAs contributed to drug resistance in OS cells. For instance, siRNA knockdown of lncRNA ANCR affects exosomal ANCR in patients with OS and doxorubicin resistance (127). These findings are expected to be applicable in clinical settings, providing an alternative treatment approach for OS.

Second, a family of short ncRNAs, called PIWI-interacting RNAs (piRNAs), with a length of approximately 30nt interacts with Piwi proteins in a functional manner. It performs biological tasks, including maintaining germline and stem cell functions, controlling translation and mRNA stability, and silencing transcribed genes (128). This type of RNA can also affect OS cell growth and metastasis. According to Das et al. (129), piRNA-39980 can activate MMP-2 and impede SERPINB1 to increase the invasion and metastasis of OS cells while suppressing their death. Wang et al. (130) also discovered notable alterations in the expression of numerous short ncRNAs, such as piRNAs, snoRNAs, and miRNAs, in MG63 OS cells using sequencing and other methods. Tumor cell growth and metastasis have been reported to be inhibited by piRNA DQ596225, snoRNA ENST00000364830.2, snRNA ENST00000410533.1, and miRNA hsa-miR-369-5p. Some medications act on piRNAs and can alter the life cycle of OS cells. Cui et al. (131) discovered that butorphanol suppresses the expression of mRNA FN1 and increases the expression of piRNA hsa_piR_006613, thereby preventing OS cell growth and metastasis. This suggests that butorphanol could serve as a novel therapeutic target for OS. Finally, a class of tiny ncRNAs called small nucleolar RNA (snoRNA) is found in the nucleolus of eukaryotic cells and is involved in the processing of mRNA3 ‘terminal and ribosomal RNA *in vivo* (132). Regarding its impact on OS cells, Xu et al. (133) discovered that lncRNA H19 suppresses the development of OS by controlling snoRNA expression. This suppression occurs by regulating snoRNA expression, including the suppression of SNORA 7A, and influencing the DNA damage response and repair protein complex. As mentioned above, snoRNA ENST00000364830.2, discovered by Wang et al. (130), can also be utilized as a biomarker for OS diagnosis. Other ncRNAs may also have an impact on OS cell growth, metastasis, and other functions; however space constraints prevent a more detailed exploration of this study. In conclusion, these ncRNAs have a significant effect on the pathophysiology of OS and hold considerable promise for use in both diagnosis and treatment.

## 8 Conclusions and future prospects

At present, there are several issues associated with primary chemotherapy and surgical treatment of OS, such as drug-resistant tumor cells and postoperative recurrence. Targeted tumor therapy has been widely pursued in the treatment of OS. Recent discoveries in the field of exosomes have led to advancements in this area. Various studies have demonstrated that ncRNA depth influences cell developmental cycles, including in OS cells. Thus, artificial manipulation of these ncRNAs for diagnosing and treating OS is a highly appropriate large entry point. However, ncRNAs are fragile in both serum and intracellular environments, and their widespread distribution poses a challenge in therapeutic applications, limiting their efficiency. Addressing these challenges could provide a valuable solution. Exosomes can be used as natural carriers of ncRNA and medications to treat OS, showing promise due to their relatively stable presence in the blood and cells and excellent tissue targeting. Previous research has focused on the function of a single ncRNA, neglecting the endogenous competitive network that ncRNAs form *in vivo*. This makes it difficult to avoid the effects of other substances *in vivo* when the research results are applied. However, progress has been made since the discovery of competitive endogenous RNA networks. In addition, the study of single RNAs in RNA networks is gradually transitioning toward exploring pathways involving multiple RNAs.

Despite these advancements, several challenges must be addressed before exosomes can effectively serve as carriers for ncRNAs in OS therapy. These challenges include difficulties in exosome enrichment, which prevents their widespread application in therapy, inadequate understanding of endogenous RNA networks to enable the development of more reliable molecular pathways that impede OS cells, and it is difficult that the carrier of loading effective ncRNAs and drugs into exosomes in a stable manner. Furthermore, there is a lack of sufficient clinical trials to confirm the efficacy of using exosomes for delivering medicines. Addressing these issues through comprehensive studies is crucial to making exosome-based therapies applicable in clinical practice, offering potential solutions to combat cancer effectively.
